# ‘How Are You Going to Make It Through?’: The Enduring Legacy of Dr. Wyona Freysteinson

**DOI:** 10.1002/nop2.70710

**Published:** 2026-07-18

**Authors:** Jodi C. Kennedy, Faith Tissot

**Affiliations:** ^1^ College of Nursing, Texas Woman's University Denton Texas USA

The death of a nurse scientist is more than the loss of an individual. It is the loss of a unique voice, a lifetime of questions yet to be explored, future discoveries left unrealized, and a mentor whose influence extends far beyond publications and classrooms. A nurse scientist's legacy is measured not only by the publications they produce or the theories they develop, but by the people they inspire, the science they advance and the generations of scholars who continue their work.

Dr. Wyona Freysteinson, PhD, RN, FAAN, dedicated her career to understanding healing, identity, embodiment and the profoundly human experience of seeing oneself after illness, trauma and visible bodily change. As her students and colleagues grieve her absence, we find ourselves living one of the very phenomena she spent her career helping others navigate: reconstructing identity after profound loss. Her scholarship taught us that healing does not erase suffering; rather, it transforms how we understand ourselves and move forward. In many ways, that lesson has become her final gift to those she mentored.

This editorial is not simply a remembrance of an extraordinary nurse scientist. It is a reflection on the enduring legacy of scholarship, the transformative power of mentorship, and the responsibility of future nurse scientists to carry forward the work of those who shape the nursing profession and the future of nursing science. History reminds us that the influence of great thinkers rarely ends with their death—Socrates left behind no written works of his own. Much of what the world knows about his philosophy survives because his student, Plato, preserved his teachings, expanded upon his ideas and carried them forward for future generations (Brickhouse and Smith 2022). In many respects, scholarship has always depended on this intergenerational transmission of knowledge. Scientific ideas endure because they are not merely published—they are taught, challenged, refined and advanced by those who inherit them.

Nursing science is no different. The true measure of a nurse scientist is not only the body of work they leave behind, but also the scholars they cultivate. Through mentorship, they instill curiosity, intellectual rigour and a commitment to advancing the discipline (Meleis et al. 1994). Their students become the custodians of unfinished questions and the architects of future discoveries.

Although death marks the end of a life, it does not mark the end of influence. For nurse scientists, scholarship becomes a voice that continues to speak through publications, theories, lectures, dissertations and, perhaps most importantly, through the scholars who question, refine and build upon their work. In this way, the death of a nurse scientist marks not the end of scholarship, but the beginning of its next chapter.

Dr. Wyona Freysteinson understood this responsibility well. She invested generously in developing nurse scientists who would not simply replicate her work but extend it into new areas of inquiry. As her students, we recognize that her legacy now rests not only in her publications or her Neurocognitive Model of Mirror Viewing but also in our willingness to continue asking the questions she taught us to ask and pursuing the discoveries she believed nursing science still had to make.

Her scientific career was devoted to understanding one of the most deeply human, yet often overlooked, aspects of health: how individuals experience themselves after illness, trauma and visible bodily change. She recognized that healing involved far more than physical recovery and challenged nursing to consider what happens when individuals no longer recognize themselves in the mirror.

Her pioneering research explored the experiences of individuals living with mastectomy, disfigurement, military sexual trauma, and other conditions that fundamentally altered body image, embodiment and identity (Freysteinson et al. 2012, 2018). Through rigorous qualitative inquiry, she illuminated the complex relationship between the physical body, personal identity and the process of healing. Rather than treating mirror viewing as a superficial or purely psychological experience, she demonstrated that it could serve as a profound encounter with the self—one in which suffering, acceptance, vulnerability, resilience and hope coexist.

This work culminated in Freysteinson's Neurocognitive Model of Mirror Viewing (Freysteinson 2020), providing nursing with a framework for understanding how individuals perceive and interpret their reflected selves. Her theory expanded nursing knowledge beyond observable symptoms to the lived experience of embodiment and identity. In doing so, she advanced nursing science by providing language and theoretical direction for phenomena that many patients experience but few clinicians had previously articulated.

Equally important was her unwavering commitment to qualitative inquiry. She demonstrated that rigorous interpretive research could generate meaningful scientific knowledge, deepen theoretical understanding and reveal dimensions of health that cannot be captured through physiological measures alone. Her scholarship affirmed that listening carefully to patients' stories is itself a scientific endeavour capable of informing theory, improving practice and advancing person‐centred care. Through her research, she reminded the nursing profession that understanding the human experience is not secondary to nursing science; it is one of its greatest strengths (Sandelowski 2004).

During our time as her doctoral students, Dr. Freysteinson often told us that one of the questions she asked her patients was, ‘How are you going to make it through?’ At first glance, the question seems simple. However, it reflects the very essence of her philosophy of nursing. Rather than telling patients what they should do or dwelling on what had been lost, she invited them to recognize their own strength and agency. The question shifted the focus from suffering to possibility, placing control back into the hands of the person experiencing illness or adversity. In doing so, she affirmed that healing is not something done to a person, but something in which the individual remains an active participant. That single question embodied how Dr. Freysteinson approached both nursing and mentorship: with unwavering faith in people's ability to persevere, grow and discover strengths they did not yet know they possessed.

She mentored her doctoral students with that same philosophy. Rather than providing answers, she taught us how to ask better questions. She challenged us to think critically, remain faithful to participants' experiences and recognize our potential long before we did. In retrospect, those moments of mentorship became more than instruction; they became part of a legacy that continues through the scholars she shaped.

Dr. Freysteinson understood that scholarship extends beyond discovery. She devoted her career not only to advancing nursing science but also to preparing future nurse scientists to continue that work. Today, her legacy endures through her publications, the Neurocognitive Model of Mirror Viewing, the dissertations she guided and the countless scholars whose work continues to build upon her own.

Perhaps this is the truest measure of a scholar's life. Scientific immortality is not found in never being forgotten, but in continuing to shape the future after one's own work has ended. By continuing to ask meaningful questions, mentor the next generation and advance the science she cared so deeply about, nurse scientists ensure that Dr. Freysteinson's voice remains an enduring part of nursing's collective story.

When a nurse scientist dies, the profession loses a voice. Whether that voice continues to shape nursing depends on those willing to preserve, challenge, refine, and extend the theories and knowledge we inherit while mentoring the scholars who will 1 day advance them. Throughout her career, Dr. Freysteinson championed the continued development of nursing theory and encouraged nurse scientists to investigate the phenomena uniquely entrusted to our profession. By investing in future generations of nurse scientists and building upon the scientific foundation left by those who came before us, we ensure that nursing science remains a living, evolving discipline. That is how nursing science endures.

## Author Contributions

**Jodi C. Kennedy:** conceptualization; writing – original draft; writing – review and editing. **Faith Tissot:** writing – review and editing; validation.

## Funding

The authors have nothing to report.

## Conflicts of Interest

The authors declare no conflicts of interest.

## Data Availability

Data sharing not applicable to this article as no datasets were generated or analysed during the current study.
